# Genetic Insights on Meropenem Resistance Concerning *Klebsiella pneumoniae* Clinical Isolates

**DOI:** 10.3390/life14111408

**Published:** 2024-11-01

**Authors:** Fathy M. Elkady, Bahaa M. Badr, Abdel-Aty E. Alfeky, Mohammed S. Abdulrahman, Amr H. Hashem, Abdulaziz A. Al-Askar, Gehad AbdElgayed, Hany R. Hashem

**Affiliations:** 1 Microbiology and Immunology Department, Faculty of Pharmacy (Boys), Al-Azhar University, Cairo P.O. Box 11884, Egypt; 2 Department of Basic Medical and Dental Sciences, Faculty of Dentistry, Zarqa University, Zarqa P.O. Box 132222, Jordan; 3 Department of Medical Microbiology and Immunology, Faculty of Medicine, Al-Azhar University (Assiut Branch), Assiut P.O. Box 71524, Egypt; 4 Botany and Microbiology Department, Faculty of Science, Al-Azhar University, Cairo P.O. Box 11884, Egypt; 5 Department of Botany and Microbiology, Faculty of Science, King Saud University, P.O. Box 2455, Riyadh 11451, Saudi Arabia; 6 Integrated Molecular Plant Physiology Research, Department of Biology, University of Antwerp, 2020 Antwerp, Belgium; 7 Department of Microbiology and Immunology, Faculty of Pharmacy, Fayoum University, Al-Fayoum P.O. Box 53514, Egypt

**Keywords:** *Klebsiella pneumoniae*, meropenem resistance, ESβL, carbapenemase, plasmid

## Abstract

The transferable genetic elements are associated with the dissemination of virulence determinants amongst *Klebsiella pneumoniae*. Thus, we assessed the correlated antimicrobial resistance in carbapenem-resistant *Klebsiella pneumoniae* clinical isolates. Each isolate’s ability to biosynthesize biofilm, carbapenemase, and extended-spectrum β-lactamase were examined. Genotypically, the biofilm-, outer membrane porin-, and some plasmid-correlated antimicrobial resistance genes were screened. About 50% of the isolates were multidrug-resistant while 98.4% were extended-spectrum β-lactamase producers and 89.3% were carbapenem-resistant. Unfortunately, 93.1% of the multidrug-resistant isolates produced different biofilm levels. Additionally, *fim*D and *mrk*D genes encoding adhesins were detected in 100% and 55.2% of the tested isolates, respectively. Also, the *bla*_KPC_, *bla*_OXA-48-like_, and *bla*_NDM_-encoding carbapenemases were observed in 16.1%, 53.6%, and 55.4% of the tested isolates, respectively. Moreover, the *bla*_SHV_ and *bla*_CTX-M_ extended-spectrum β-lactamase-associated genes were detected at 95.2% and 61.3%, respectively. Furthermore, *aac(3)IIa*, *qnr*B, and *tet*B resistance-correlated genes were observed in 38.1%, 46%, and 7.9% of the tested isolates, respectively. Certainly, the tested antimicrobial resistance-encoding genes were concurrently observed in 3.2% of the tested isolates. These findings confirmed the elevated prevalence of various antimicrobial resistance-associated genes in *Klebsiella pneumoniae*. The concurrent transferring of plasmid-encoding antimicrobial resistance-related genes could be associated with the possible acquisition of multidrug-resistant *Klebsiella pneumoniae* phenotypes.

## 1. Introduction

Multidrug-resistant (MDR) *Klebsiella pneumoniae* (*K. pneumoniae*) phenotypes are more complicated with extended-spectrum β-lactamases (ESβL) production resulting in management failure accompanied by a high rate of mortality. The production of more than 300 ESβL variants is the major β-lactam-inactivating mechanism amongst *K. pneumonia* clinical isolates [1]. These enzymes break down the β-lactam ring in penicillins, most cephalosporins (1^st^, 2^nd^, 3^rd^, and 4^th^ generations), and monobactams. Fortunately, these enzyme-hydrolyzing effects could be evaded by sulbactam, tazobactam, or clavulanic acid. Accordingly, such ESβL inhibitory action expects their benefits in controlling and the phenotypic identification of *K. pneumoniae* isolates producing EsβLs [2,3]. The β-lactamase sulfhydryl variable active site (*bla*_SHV_), β-lactamase Temoneira (*bla*_TEM_), and β-lactamase cefotaximase (*bla*_CTX-M_) genes are commonly detected in *K. pneumoniae*. These genes encode the Ambler class A ESβLs and are transferred via conjugative plasmid [4]. On top, the increased prevalence and diversity of *bla*_CTX-M_ diminish the usefulness of third generation cephalosporins in the treatment of serious *K. pneumoniae* infections [5].

The ESβLs have no hydrolyzing activity on cephamycins and carbapenems, which clarifies their current use in controlling infections caused by ESβLs producing *K. pneumoniae* [6]. Unfortunately, recent carbapenem’s extensive misuse has resulted in increasingly recorded worldwide *K. pneumoniae*-resistant strains via Ambler class B zinc-dependent β-lactamase enzymes [7]. The production of carbapenemase encoded by the *bla*_KPC_ gene is the major mechanism of resistance in the carbapenem-resistant *K. pneumoniae* (CRKp) [8]. Also, *K. pneumoniae* cell membrane structural modifications inhibit carbapenems entering the cells. This modification results from the alterations or complete loss of the non-selective major porins, OmpK35 and OmpK36 [9]. The presence of plasmid-mediated genes encoding resistance determinants is usually associated with the mutational loss of the bigger and more leaky membrane channels encoded by *omp*K35 and *omp*K36 genes in CRKp. This is due to the counteraction of the β-lactamase hydrolyzing effect via the fast and continuous β-lactam influx into the bacterium cell through the normal large-pore protein channels with the end result of low levels of resistance [10].

Also, the production of N-6′-acetyltransferase-Ib enzyme encoded by the plasmid-mediated *aac(3)IIa* gene in *K. pneumoniae* isolates results in the modified inactivation of aminoglycosides [11]. Additionally, the *qnr* plasmid-mediated gene is responsible for the inactivation of fluoroquinolones in *K. pneumoniae*-resistant isolates via bacterial gyrase protection [12,13]. The frequent co-existence of such transferable plasmid-mediated resistance genes is accountable for the emergence of ESβLs and carbapenemase-producing *K. pneumoniae* with fluoroquinolone and/or aminoglycoside resistance [14]. Moreover, a serious distribution of antimicrobial resistance (AMR) amongst diverse microorganisms is generated by the acquisition of these plasmid-mediated transferable genes [15].

Moreover, the colonization, invasion, and pathogenesis of *K. pneumoniae* strains are associated with several virulence factors. Among these is the successful attachment to host cells by bacterial fimbriae encoded by *fim*D and *mrk*D genes with the subsequent enhancement of bacterial biofilm formation [16,17]. Alarmingly, the disorders triggered by biofilm-forming MDR *K. pneumoniae* in some individuals are usually associated with a high mortality rate [18]. Hence, this study aimed to determine the AMR profile, biofilm formation capability, and incidence of plasmid-mediated resistance genes in ESβL-producing CRKp clinical isolates from hospitalized patients.

## 2. Materials and Methods

### 2.1. Presumptive Identification of K. pneumoniae Isolates

Three hundred and seventeen clinical specimens including blood (n = 54), urine (n = 65), sputum (n = 85), pus of abscesses (n = 69), and ear discharge (n = 44) were collected from inpatients attending Al-Azhar University hospitals, Cairo, Egypt, between November 2022 and August 2023. The phenotypic identification of *K. pneumoniae* isolates was performed depending on their cultural characteristics on MacConkey’s agar, triple sugar iron (TSI) agar, motility, indole, and ornithine decarboxylation (MIO) media (Oxoid, Basingstoke, UK). Moreover, microscopical examination, Gram and capsular staining, and biochemical behavior including the production of oxidase, catalase, urease, and citratase enzymes were performed according to [19,20].

### 2.2. Antimicrobial Susceptibility Testing

The antimicrobial susceptibility of the *K. pneumoniae* isolates was assessed using the resazurin-based broth microdilution method following the Clinical and Laboratory Standards Institute guidelines [21]. Diverse antimicrobial agent discs (Oxoid, Basingstoke, UK) representing different classes were tested including cephalosporins (ceftriaxone, cefotaxime, and ceftazidime), carbapenem (meropenem), β-lactam/clavulanic acid combination (amoxicillin/clavulanic), aminoglycosides (amikacin and gentamycin), fluoroquinolone (ciprofloxacin), and tetracyclines (doxycycline and tetracycline). Briefly, a pure 0.5 McFarland standard of each tested isolate suspended in tryptic soya broth (TSB) (Oxoid, Basingstoke, UK) was prepared. One hundred microliters of each tested agent stock solution (512 μg/mL) were added to the 1^st^ well of each row and TSB was inoculated as 50 μL/well into the remaining wells. Fifty microliters of each stock solution in the 1st well were transferred into the 2nd well, and this step was repeated from the 2nd well to the 3rd well till the 12th well. The prepared bacterial suspension (50 μL) was then added to each well followed by the addition of 100 μL of TSB. The wells containing live bacterial cells without any tested compound and TSB without cells were included in each plate as positive and negative controls, respectively. The plates were incubated at 37 °C for 18 h, and 40 μL of resazurin (0.015%) was then added to each well followed by incubation for another 2 h. The lowest concentration of each tested agent at which no bacterial growth was observed, with consequent unchanged blue resazurin color, was scored as the MIC [22]. The results were interpreted as resistant, intermediate, or susceptible and were conducted according to the breakpoints specified by [21]. The *K. pneumoniae* isolates resistant to at least one agent in three or more antimicrobial classes were recorded as MDR [23].

### 2.3. Phenotypic Screening of ESβL and Carbapenemase Production

The combined disc test was performed for the phenotypic confirmation of *K. pneumoniae*-ESβL producers. Briefly, the tested isolate suspension, equivalent to 0.5 McFarland turbidity standard, was inoculated onto the surface of a Mueller–Hinton agar (MHA) (Oxoid, Basingstoke, UK) plate using a cotton-tipped sterile swab. Cefotaxime (30 µg) disc (Oxoid, Basingstoke, UK) was placed on the agar surface at 25 mm (center to center) from the cefotaxime/clavulanic acid (30/10 µg) disc (Oxoid, Basingstoke, UK). The plate was then incubated at 37 °C and the inhibition zones were determined after 24 hrs. The combined disc showing an inhibition zone of ≥5 mm compared with the single disc was indicative of synergistic interaction between cefotaxime and clavulanic acid and proposed the ESβL-producing phenotype of the tested isolate [2].

Additionally, carbapenemase production was assessed phenotypically following the modified carbapenem inactivation method (MCIM) as described by [24]. Briefly, bacterial colonies from overnight culture on blood agar were inoculated into 2 mL TSB, and an imipenem disc (10 μg) (Oxoid, Basingstoke, UK) was then immersed in the prepared bacterial suspension. After incubation at 37 °C for 4 h, the imipenem disc was removed using a sterile inoculation loop and firmly located on an MHA plate inoculated with imipenem-sensitive *E. coli* ATCC 25,922 strain, followed by incubation for 24 h at 37 °C. The growth of imipenem-sensitive *E. coli* ATCC 25,922 strain around the treated imipenem disc at a zone diameter of 6–15 mm or the appearance of bacterial colonies within a zone diameter of 16–18 mm reflects the ability of the tested isolate to produce carbapenemases that hydrolyze the imipenem. On the other hand, if the treated imipenem disc showed an inhibition zone diameter ≥19 mm, it indicated intact imipenem and the inability of the tested bacterial isolates for carbapenemase production.

### 2.4. Quantitative Estimation of Biofilm Formation

The ability of the tested *K. pneumoniae* isolates to form biofilm was quantitatively estimated using the microtiter plate assay as previously described by [25]. Briefly, several colonies of each bacterial isolate were inoculated into TSB supplemented with 1% glucose (TSBG) followed by incubation at 37 °C for 24 h. Each bacterial culture of 0.5 McFarland standard (1.5 × 10^8^ CFU/mL) was then diluted 1:100 using sterile TSBG. In each well of the sterile 96-well microtiter plates, 200 μL of the prepared bacterial culture was added and incubated at 37 °C for 24 h. The sterile TSBG was used as a negative control (NC). The broth was then removed from each well followed by washing four times using 200 μL phosphate-buffered saline (PBS) at pH 7.2 and desiccation at 60 °C for 1 h. The remaining bacterial biofilms were then stained with 2% crystal violet for 15 min and the excess stain was discarded followed by washing three times with PBS. In each well, the bound dye was dissolved in 180 μL of 33% (*v*/*v*) glacial acetic acid. The assays were carried out in triplicate and the absorbance or optical density (OD) for each well was quantified at 570 nm (OD_570_). For each assay, the mean OD and standard deviation (SD) for the NC were determined and the cut-off optical density (OD_c_) was calculated according to the formula OD_c_ = mean OD of NC + 3 SD of NC. Bacterial isolates were scored as non-biofilm forming when their mean OD (OD_t_) ≤ OD_c_, weakly biofilm forming (+) when OD_c_ < OD_t_ ≤ 2 OD_c_, moderately biofilm forming (++) when 2 OD_c_ < OD_t_ ≤ 3 OD_c_, or strongly biofilm forming (+++) when 3 OD_c_ ≥ OD_t_.

### 2.5. Molecular Identification of the Tested Isolates

The definite identification of *K. pneumoniae* isolates based on the polymerase chain reaction (PCR) amplification of the conserved 16S−23S rDNA internal transcribed spacer (ITS) sequence. Briefly, total bacterial DNA was extracted following the boiling method previously described by [26] and specific F/R_1_ and F/R_2_ primers (Table 1) were used according to [27]. Each PCR mixture contained 12.5 μL Cosmo PCR Red Master Mix (Willowfort, Birmingham, UK), 50 ng genomic DNA, 1 μL of each primer, and nuclease-free water to 25 μL total volume. The PCR mixture containing nuclease-free water instead of the bacterial DNA or *K. pneumoniae* ATCC 13,882 standard strain DNA was used as a negative or positive control, respectively. Each target sequence was amplified following the program shown in Table 2 using a Thermal cycler (Biometra UNO–Thermoblock, Gottingen, Germany). The PCR amplification products were then detected with the aid of 1.5% agarose gel stained with ethidium bromide (0.05 mg/mL). Electrophoresis was conducted in Tris-acetate EDTA buffer for 45 min (Sigma Aldrich, Hamburg, Germany) at 100 V, and a UV transilluminator (HERMLE Labortechnik GmbH, Wehingen, Germany) was then used to visualize the PCR products. The specific band molecular size was determined by comparing it with a 100 bp DNA marker (Geneaid Biotech Ltd., New Taipei City, Taiwan) as a suitable ladder [28].

### 2.6. Molecular Detection of Biofilm-, ESβL-, Carbapenemase-, and Other AMR-Related Genes

In the tested *K. pneumonia* isolates, the existence of genes encoding *fim*D and *mrk*D fimbrial adhesion-associated genes was evaluated using the PCR technique [29,30]. Additionally, the presence of various genes responsible for AMR was monitored. The prevalence of ESβLs-associated genes, *bla*_SHV_ and *bla*_CTX-M_, was verified according to the method described by [31]. Moreover, the *bla*_KPC_, *bla*_OXA-48-like_, and *bla*_NDM_ carbapenemase-encoding genes were amplified [32,33]. Moreover, the incidence of *omp*K35 and *omp*K36 genes, encoding for porins that are responsible for decreased outer membrane permeability to carbapenems, was estimated [23]. Furthermore, the occurrences of the aminoglycoside resistance-related gene, *aac(3)IIa* [34]; the quinolone resistance-encoding gene, *qnr*B [26]; and the tetracyclines resistance-associated gene-encoding efflux pump, *tet*B were investigated [35].

Each singleplex PCR mixture for each amplified gene contained 12.5 μL Cosmo PCR Red Master Mix (Willowfort, Birmingham, UK), 50 ng of the extracted DNA, 1 μL of each specific primer for the target gene amplification (Table 1), and nuclease-free water to 25 μL total volume. A negative control reaction mixture containing no DNA was included.

The PCR amplification programs were adjusted at the conditions suitable for each amplified gene (Table 2). Each PCR amplification product was separated by electrophoresis as previously described by [28].

**Table 1:** PCR oligonucleotide primers target specific sequences in *K. pneumoniae* isolates.

Target Genes	Primer Sequence (5′ → 3′)	Amplicon Size (bp *)	References
16S−23S rDNA	F*-ATTTGAAGAGGTTGCAAACGAT	130; F/R_1_ 260; F/R_2_	[27]
	R*_1_-TTCACTCTGAAGTTTTCTTGTGTTC		
	R_2_-CCGAAGATGTTTCACTTCTGATT		
*fim*D	F; GTTACGCCTATCTGAATCTACAGAG	1114	[30]
	R; GACCAGTTGATATCGTCCACG		
*mrk*D	F; CGCTTTTTATCGTCTTAATG	880	
	R; GTGATGTAGCGGGTCTCCTG		
*bla*_NDM_	F; GCAGCTTGTCGGCCATGCGGGC	782	[32]
	R; GGTCGCGAAGCTGAGCACCGCAT		
*bla*_KPC_	F; CGTCTAGTTCTGCTGTCTTG	798	[33]
	R; CTTGTCATCCTTGTTAGGCG		
*bla*_OXA-48-like_	F; GCGTGGTTAAGGATGAACAC	438	
	R; CATCAAGTTCAACCCAACCG		
*omp*K35	F; ATGATGAAGCGCAATATTCTGGCAGTGG	684	[23]
	R; R; TCGGCTTTGTCGCCATTGCCGTCA		
*omp*K36	F; ATGAAAGTTAAAGTACTGTCCCTC	1076	
	R; GTCGTCGGTAGAGATACCGGC		
*bla*_SHV_	F; TCCCATGATGAGCACCTTTAAA	104	[31]
	R; TCCTGCTGGCGATAGTGGAT		
*bla*_CTX-M_	F; TCTTCCAGAATAAGGAATCCC	909	
	R; CCGTTTCCGCTATTACAAAC		
*aac(3)IIa*	F; ATATCGCGATGCATACGCGG	877	[34]
	R; GACGGCCTCTAACCGGAAGG		
*qnr*B	F; GATCGTGAAAGCCAGAAAGG	469	[26]
	R; ACGATGCCTGGTAGTTGTCC		
*tet*B	F; CAGTGCTGTTGTTGTCATTAA	571	[35]
	R; GCTTGGAATACTGAGTGTAA		

* bp, base pair; F, forward; R, reverse.

**Table 2:** PCR amplification conditions of each target sequence.

Target Genes	PCR Conditions			References
	Initial Denaturation	Elongation (35 Cycles)	Final Extension	
16S-23S rDNA	94 °C/10 min	94 °C/30 s, 57 °C/20 s, and 72 °C/20 s	72 °C/10 min	[27]
*fim*D	95 °C/5 min	95 °C/30 s, 57 °C/60 s, and 72 °C/60 s		[30]
*mrk*D		95 °C/30 s, 55 °C/60 s, and 72 °C/60 s		
*bla*_NDM_	95 °C/5 min	95 °C/45 s, 60 °C/45 s, and 72 °C/60 s	72 °C/8 min	[32]
*bla*_KPC_		95 °C/30 s, 60 °C/90 s, and 72 °C/60 s	72 °C/10 min	[33]
*bla*_OXA-48-like_		95 °C/30 s, 56 °C/30 s, and 72 °C/60 s		
*omp*K35	93 °C/3 min	93 °C/60 s, 55 °C/60 s, and 72 °C/60 s	72 °C/7 min	[23]
*omp*K36				
*bla*_SHV_	95 °C/5 min	95 °C/30 s, 50 °C/30 s, and 72 °C/60 s	72 °C/5 min	[31]
*bla*_CTX-M_		95 °C/30 s, 60 °C/30 s, and 72 °C/60 s		
*aac(3)IIa*	95 °C/5 min	95 °C/60 s, 56 °C/30 s, and 72 °C/60 s	72 °C/5 min	[34]
*qnr*B	94 °C/5 min	94 °C/30 s, 53 °C/45 s, and 72 °C/45 s	72 °C/5 min	[26]
*tet*B		94 °C/30 s, 50 °C/45 s, and 72 °C/45 s	72 °C/10 min	[35]

### 2.7. Statistical Analysis

The analysis of variance (ANOVA) and significance (P) of the data were performed with the GraphPad Prism^®^ Software, version 8.0.2 Statistical Package. In addition, statistical correlation coefficients between the different variants were conducted using the Pearson correlation coefficient with the Statistical Package for the Social Sciences (SPSS) Software, version 25. For all the comparisons, statistical significance was considered with a *p*-value < 0.05. The antimicrobial resistance profiles and the biofilm formation results are displayed as descriptive data of relative frequencies and percentages.

## 3. Results

### 3.1. Phenotypic Identification of Isolates

A total of sixty-three *K. pneumoniae* identified isolates were recovered from different clinical sources including sputum (n = 20; 31.7%), urine (n = 17; 27%), pus of abscesses (n = 15; 23.8%), blood (n = 6; 9.6%), and ear discharge (n = 5; 7.9%). The isolates with large pink mucoid colonies on MacConkey’s agar after 24 h incubation at 37 °C with oxidase negative reaction were initially suspected as *K. pneumoniae*. They appeared as Gram-negative, capsulated, non-motile rods. Biochemically, these isolates were able to produce citratase, urease, and catalase, while they were negative for indole and ornithine decarboxylase tests. Moreover, all the *K. pneumoniae* isolates grown on TSI showed acidic slant and butt with H_2_ gas production but failed to produce H_2_S gas.

### 3.2. Antimicrobial Susceptibility Profile

The antimicrobial susceptibility testing recorded a relatively high resistance pattern among the tested *K. pneumoniae* isolates (Table 3). The frequency of the MDR *K. pneumoniae* isolates was 46% (29/63). Concerning the β-lactams, the majority of these isolates were resistant to ceftriaxone and cefotaxime at a rate of 84.1% (53/63) and 82.5% (52/63), respectively. Additionally, 50 (79.4%) and 44 (69.8%) of the isolates were resistant to amoxicillin/clavulanic and ceftazidime, respectively. A high resistance rate of the tested isolates was also observed against meropenem at 54% (34). Fortunately, low resistance patterns with a rate of 20.6% (13/63) and 23.8% (15/63) were demonstrated regarding tetracycline and doxycycline, respectively.

### 3.3. Phenotypic Screening of the ESβL and Carbapenemase Production

**Figure 1:**
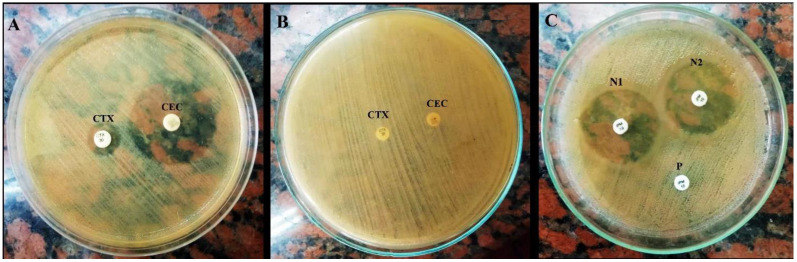
ESβL-positive (**A**) and -negative (**B**) phenotypes. CTX: cefotaxime and CEC: cefotaxime/clavulanic acid. Phenotypic carbapenemase activity (**C**). P: positive phenotype and N_1_ and N_2_: negative phenotypes of *K. pneumoniae* isolates.

The ESβL-producing phenotype was ubiquitous among the tested *K. pneumoniae* isolates. From the broth microdilution method, 98.4% (62/63) of the tested isolates were resistant to either ceftriaxone, cefotaxime, or ceftazidime and consequently were considered ESβL producers. The β-lactams protection activity of clavulanic acid, in a combined disc test (Figure 1A,B), confirmed the phenotypic ESβL production capability in all of these isolates.

Concerning the broth microdilution test, of the 63 tested *K. pneumoniae* isolates, 34 (54%) and 22 (34.9%) isolates were phenotypically resistant and intermediately resistant to meropenem, respectively. Among these carbapenem-resistant isolates, 89.3% (50/56) showed positive MCIM (Figure 1C). The results also showed that all of these isolates (50/56; 89.3%) showed both ESβL and carbapenemase production.

### 3.4. Biofilm Formation Characters Amongst K. pneumoniae Phenotypes

**Figure 2:**
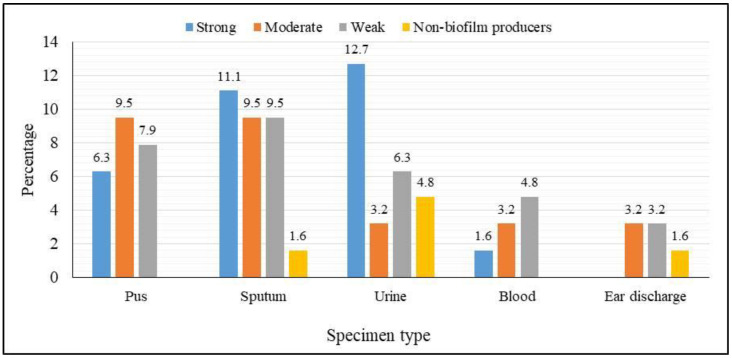
Biofilm formation ability of *K. pneumoniae* isolates regarding each type of clinical specimen.

Investigating the biofilm formation ability, the majority of the *K. pneumoniae* isolates (92.1%; 58/63) were biofilm producers with a variable degree. They were categorized by the microplate assay into weak (34.5%; 20/58), moderate (31%; 18/58), and strong (34.5%; 20/58) biofilm producers. Conversely, only 5/58 (7.9%) isolates were non-biofilm producers. The biofilm formation ability of the *K. pneumoniae* isolates in relation to each type of clinical specimen exhibited that the isolates recovered from sputum have the highest ability to form biofilm as illustrated in Figure 2. Phenotypically, the tested *K. pneumoniae* isolates showed a high AMR rate for most of the tested antimicrobial agents. The statistical analysis of these findings using the Pearson correlation coefficient revealed a positive correlation (*r* = 0.43) between biofilm formation capability and the AMR in this study isolates; however, this correlation was statistically insignificant (*p* = 0.99).

Additionally, a total of 29 (46%) were MDR *K. pneumoniae* isolates, of which 27 (93.1%) were biofilm producers. Regarding the biofilm formation categories, 12/29 (41.4%), 6/29 (20.7%), and 9/29 (31%) of the MDR isolates were strong, moderate, and weak biofilm producers, respectively, while 2/29 (6.9%) of the MDR isolates were non-biofilm producers. Indeed, there was a surprisingly statistically positive correlation (r); moreover, this correlation showed a high statistical significance (*p* = 0.008). 

### 3.5. Molecular Identification K. pneumoniae Isolates

**Figure 3:**
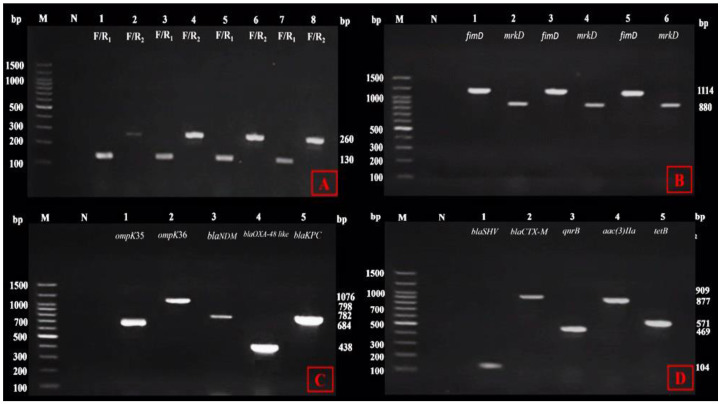
Agarose gel electrophoresis showing gene-specific PCR products from the *K. pneumoniae* isolates. (**A**) The 16S–23S rDNA. Lanes from 1 to 6: species-specific positive amplicon results for representative tested isolates; Lanes 7 and 8: positive control using DNA from *K. pneumoniae* ATCC 13,882 standard strain according to the presence of F/R_1_ (130 bp) and F/R_2_ (260 bp), respectively. (**B**) Biofilm-related genes, *fim*D (1114 bp) and *mrk*D (880 bp). Lanes from 1 to 6: positive biofilm-forming *K. pneumoniae* isolates. (**C**) The outer membrane porins (OMPs) and carbapenemase-encoding genes. Lanes from 1 to 5: specific bands of *omp*K35 (684 bp), *omp*K36 (1076 bp), *bla*_NDM_ (782 bp), *bla*_OXA-48-like_ (438 bp), and *bla_KPC_* (798 bp) in carbapenemase-producing *K. pneumoniae* isolates. (**D**) The ESβLs [*bla*_SHV_ (104 bp) and *bla*_CTX-M_ (909 bp)], *qnr*B (469 bp), *aac(3)IIa* (877 bp), and *tet*B (571 bp) genes. Lanes from 1 to 5: specific bands of *bla*_SHV_, *bla*_CTX-M_, *qnr*B, *aac(3)IIa*, and *tet*B in representative MDR *K. pneumoniae* isolates. bp, base pair; Lane M, 100 bp DNA ladder; Lane N, negative control.

Genotypically, all the tested *K. pneumoniae* isolates (100%, 63) were definitely identified. They showed a species-specific PCR product of the 16S-23S rDNA internal transcribed spacer sequence with an amplicon size of either 130 bp or 260 bp depending on the presence of either F/R_1_ or F/R_2_ primers, respectively (Figure 3A).

### 3.6. Molecular Detection of the Biofilm-Associated Genes

The biofilm-associated genes, *fim*D and *mrk*D, were detected in 58/58 (100%) and 32/58 (55.2%) of the biofilm producers, respectively (Figure 3B), where 20 (100%) and 16 (80%) of the strong biofilm producers, 18 (100%) and 9 (50%) of the moderate biofilm producers, and 20 (100%) and 7 (35%) of the weak biofilm producers harbored *fim*D and *mrk*D, respectively. These genes were simultaneously detected in 32/58 (55.2%) of the tested *K. pneumoniae* isolates, though 26/58 (44.8%) of the isolates harbored at least one of these two genes. Conversely, the *fim*D and *mrk*D genes were not detected in the phenotypically detected non-biofilm-forming *K. pneumoniae* (5/63; 7.9%). Our observations illustrated the statistically significant correlation (r) between the existence of the *fim*D (*p* = 0.01) and *mrk*D genes (*p* = 0.03) and the biofilm formation in the tested *K. pneumoniae* isolates.

### 3.7. Detection of Carbapenemase-, ESβL- and Other Plasmid-Mediated Resistance Genes

According to the broth microdilution method and the positive results of MCIM, the majority of the tested isolates (56/63; 88.9%) are characterized by a variable degree of phenotypic carbapenem resistance. On the genetic side, the *bla*_KPC_, *bla*_OXA-48-like_, and *bla*_NDM_ genes were detected in 16.1% (9/56), 53.6% (30/56), and 55.4% (31/56) of the tested CRKp isolates, respectively (Figure 3C). The results demonstrated the simultaneous finding of these genes in 3/56 (5.3%) of the CRKp isolates, while 41/56 (73.2%) isolates carried one or more of these genes. On the other hand, none of these carbapenemase-encoding genes were detected in 28.6% (12/56) of the CRKp isolates. The statistical analysis of the previous results confirmed the positive correlation (r) between the occurrence of the *bla*_KPC_, *bla*_OXA-48-like_, and *bla*_NDM_ genes and phenotypic meropenem resistance in this study isolates. Moreover, the correlation was only statistically significant in the case of the *bla*_NDM_ gene (*p* = 0.02).

Relating to carbapenem resistance, the *omp*K35 and *omp*K36 genes were frequently detected in 51/56 (91.1%) and 50/56 (89.3%) of the CRKp isolates, respectively (Figure 3C). Both genes were concurrently present in 85.7% (48/56) of the tested CRKp isolates, while 8.9% (5/56) of these isolates harbored at least one of these genes. The *omp*K35 and *omp*K36 genes were not observed in only 5.4% (3/56) of the tested isolates and were concurrently observed in 4/7 (57.1%) of the carbapenem-susceptible isolates. In our *K. pneumoniae* isolates, the statistical correlation (r) between the presence of the *omp*K35 and *omp*K36 genes and phenotypic carbapenem resistance was positive. Notably, these correlations were statistically significant with *p* = 0.02 and 0.01 for both genes, respectively. 

The *bla*_SHV_ and *bla*_CTX-M_ ESβL-encoding genes were observed in the phenotypically identified ESβL-producing *K. pneumoniae* isolates at rates of 95.2% (59/62) and 61.3% (38/62), respectively (Figure 3D). At least one of these genes was detected in 43.5% (27/62) of the tested isolates, while 56.4% (35/62) of the isolates harbored both genes. In contrast, the only phenotypically non-ESβL-producing *K. pneumoniae* isolate did not carry any of these genes. Statistically, the correlation coefficient (r) for the presence of the *bla*_SHV_ and *bla*_CTX-M_ genes and ESβL production in the *K. pneumoniae* isolates were positive in both genes, but these correlations were statistically insignificant with *p* = 0.22 and 0.06 for both genes, respectively.

Likewise, screening for the *aac(3)IIa* gene illustrated its reasonable prevalence at the rate of 38.1% (24/63) among the tested *K. pneumoniae* isolates, of which 23/24 (95.8%) isolates were identified phenotypically as aminoglycoside-resistant (Figure 3D). A total of 34 *K. pneumoniae* isolates were resistant to aminoglycosides with a percentage of 53.9%. Upon the molecular analysis of the *aac(3)IIa* gene among these isolates, the gene was detected in 13 (38.2%) isolates only. Statistical analysis revealed a positive correlation value (r); however, it was statistically insignificant (*p* = 0.75).

The *qnr*B gene was observed at a moderate rate (46%; 29/63) (Figure 3D). The detection of this gene in 24/30 (80%) of ciprofloxacin-resistant *K. pneumoniae* isolates illustrated its statistically significant correlation (*p* = 0.03) with ciprofloxacin inactivation in this study. On the other hand, a low rate (7.9%; 5/63) of the *tet*B gene was detected in the tested *K. pneumoniae* isolates (Figure 3D). The observation of the *tet*B gene in 5/15 tetracycline-resistant isolates (*p* = 0.85) indicated its insignificance correlation with tetracycline resistance in our isolates.

A substantial co-existence of various plasmid-mediated resistance genes was noted in the tested *K. pneumoniae* isolates, indicating a possible acquisition of bacterial MDR phenotype due to the concurrent transferring of several plasmid-mediated resistance genes. This co-occurrence has classified the *K. pneumoniae* isolates into five categories which were classified arbitrarily from A to E, where 2 (3.2%) isolates simultaneously carried ESβL-, carbapenemase-, aminoglycoside resistance-, quinolone resistance-, and tetracycline resistance-encoding genes (category A); 11 (17.4%) isolates concurrently carried ESβL-, carbapenemase-, aminoglycoside resistance-, and one of quinolone or tetracyclines resistance-encoding genes (category B); and 22 (35%) contained at least 3 genes of the tested classes (category C) (Table 4).

## 4. Discussion

*K. pneumoniae* represents one of the major infectious agents causing nosocomial and community-acquired microbial infections. The spreading of their AMR is associated with transferable genetic elements that may carry other virulence factors [36]. Thus, we investigated the incidence of plasmid-mediated resistance amongst CRKp isolated in diverse infections from hospitalized patients at tertiary hospitals in Cairo, Egypt.

Our analysis recorded a slightly high incidence (46%; 29/63) of the MDR *K. pneumoniae* phenotype with elevated resistance profiles towards β-lactams, where 54% (34/63) of the tested isolates were resistant to meropenem. In a comparable study, an equal rate (46%; 43/93) of MDR *K. pneumoniae* strains was obtained [37]. Moreover, a related study in Sudan recorded a much higher rate (80%) of MDR profiles and a relatively similar rate (51%) of carbapenem resistance [38]. This high rate of MDR phenotype and carbapenem resistance observation may be attributed to the continuous development of MDR strains due to the extensive and/or misuse of antimicrobial agents worldwide.

The spreading of ESβLs has been recognized as a foremost global health concern. Consequently, this study focused on the phenotypic detection of ESβL production. The tested *K. pneumoniae* isolates which showed reduced susceptibility to either cefotaxime, ceftriaxone, or ceftazidime were initially assumed to be an ESβL producer. The findings illustrated the ubiquitous nature of ESβLs (98.4%; 62/63) among the identified *K. pneumoniae* clinical isolates. In the studies carried out by [39] and [37], 36 (40%) and 60% (56/93) of their tested *K. pneumoniae* strains were phenotypically ESβL producers, respectively. The widespread use of third generation cephalosporins may clarify the cause of higher ESβL production rates amongst our tested *K. pneumoniae* isolates. 

The appropriate diagnosis of the infections caused by CRKp isolates, a high risk to human health, will help to find an effective treatment for patients. Phenotypically, this study’s findings showed a high rate (88.9%; 56/63) of CRKp. Moreover, 89.3% (50/56) of the isolates showed a positive MCIM and all of them were phenotypically ESβL producers. In a related study, the MCIM showed 95.4% sensitivity and 100% specificity for detecting CRKp [40]. These results illustrated the importance of MCIM for phenotypic carbapenemase detection. This reliable, simple, and inexpensive test can be performed with the usual antimicrobial susceptibility testing for the rapid identification of CRKp isolates. 

Biofilm formation is one of the most significant bacterial virulence features. It is considered a key step in *K. pneumoniae* pathogenesis. The aggregation of these bacteria within the biofilm structure is difficult to be eradicated as it protected from antimicrobial agents and the host’s immune response. In the present study, the tested *K. pneumoniae* isolates were mostly biofilm producers (92.1%; 58/63), where 34.5% (20/58) were strong biofilm producers. Also, the majority (93.1%: 27/29) of the MDR isolates were capable of biofilm formation. Similarly, biofilm production was observed in all of the tested *K. pneumoniae* isolates with 57% strong biofilm-forming phenotype [41]. A related high rate (80%; 36/45) of *K. pneumoniae* biofilm formation was also recorded but with a low level of strong biofilm-forming category [42]. Another study reported a lower frequency (44.4%) of biofilm production without a strong biofilm-forming phenotype in *K. pneumoniae* isolates [43]. This variation in biofilm formation capacity may be explained by the variation in sample size, specimen type, adhesive characters of the tested strains, sensitivity of the applied method, and diverse extent of the biofilm-associated genes.

Biofilm-forming *K. pneumoniae* isolates mostly carry fimbrial adhesins encoding genes. In the present study, the occurrence of selected considerable virulence genes encoding fimbriae was investigated. The *fim*D, encoding type 1 fimbriae, was detected in all *K. pneumoniae* biofilm producers (100%), while *mrk*D encoding type 3 adhesin was detected only in 55.2%. Simultaneous detection of these genes was observed in 32/58 (55.2%) of the tested *K. pneumoniae* isolates. In a related study performed by [30], *fim*D and *mrk*D genes were concurrently presented in 98.43% of their tested isolates with higher *mrk*D incidence. Also, *mrk*D was frequently identified in 96.3% and 88.5% of *K. pneumoniae* isolates as reported by [44,45], respectively. These differences in such virulence gene detection rates may reflect the variation in molecular mechanisms involved in adhesion, colonization, and bacterial biofilm formation. 

Globally, the persistent- and misuse of carbapenems results in an elevated prevalence of CRKp. Hydrolysis by carbapenemases represents the main abrogation mechanism in CRKp. In this study, the presence of three of the most important carbapenemase-related genes, *bla*_NDM_, *bla*_oxa-48-like_, and *bla*_KPC_, was inspected, with detection rates of 55.4%, 53.6%, and 16.1%, respectively. In a similar study, all the tested *K. pneumoniae* were CRKp and carbapenemase producers. The commonly detected carbapenemase genes were *bla*_NDM_ (72.3%) and bla_KPC_ (24.5%) [46]. Also, higher *bla*_oxa-48-like_ and *bla*_NDM_ rates of 90.1% and 70.5%, respectively, were observed in the study conducted by [47] and the *bla*_oxa-48-like_ gene was the most abundant. Accordingly, continuous screening for genes responsible for carbapenem resistance in CRKp is a crucial factor in avoiding the emergence of carbapenemase-producing phenotype associated with the consumption of these antimicrobial agents.

The loss of OMPs due to OmpK35- and OmpK36-encoding gene down-regulation or point mutations is a complementary carbapenem resistance mechanism in *K. pneumoniae* strains. In the current study of CRKp isolates, the *omp*K35 and *omp*K36 genes were widely observed at the rates of 91.1% and 89.3%, respectively. The majority of the tested isolates (85.7%) were concurrently carried both *omp*K35 and *omp*K36 genes. Similarly, in the study carried out by [48], *omp*K35 and *omp*K36 were identified in 82% and 76% of the CRKp isolates, respectively. However, in the current work, there is an insignificant correlation (*p* = 0.13) between the presence of both genes and the reduced susceptibility to meropenem.

*K. pneumoniae* isolates producing carbapenemase and ESβL are usually associated with multiple antimicrobial agents’ resistance. They represent frequent trouble with limited therapeutic options. This study’s findings illustrated the detection of both or at least one of the *bla*_SHV_ or *bla*_CTX-M_ genes among the phenotypically ESβLs producing *K. pneumoniae* isolates. These genes were observed in 95.2% and 61.3% of the tested isolates, respectively. Likewise, the study conducted by [49] reported that *bla*_SHV_ was the most prevailing ESβL gene in *K. pneumoniae*. However, this gene was the second most commonly detected ESβL gene at a rate of 98.7% [47]. In another study, two ESβL genes, *bla*_CTX-M_ and *bla*_SHV_, were detected in two *K. pneumoniae* isolates and three ESβL genes, *bla*_CTX-M_, *bla*_SHV_, and *bla*_TEM_, were simultaneously observed in only one isolate [39]. These results illustrate the notable frequency of the *bla*_SHV_ ESβL gene as a molecular resistance mechanism. However, different geographical locality may explain the variable incidence of ESβL genes.

Aminoglycoside modifications, due to genes encoding aminoglycoside-modifying enzymes, resulted in their reduced binding affinity to the target site with subsequent loss of antibacterial activities. In the current study, the *aac(3)IIa* gene was predominantly observed in the phenotypically identified aminoglycosides-resistant *K. pneumoniae* isolates (95.8%; 23/24). In previous studies, this gene was also detected with reduced susceptibility to aminoglycosides, but at much lower rates, 27% [50] and 21.2% [34] which could be explained by the presence of other genetic determinants for aminoglycoside resistance.

The modification of Qnr proteins, encoded by *qnr* plasmid-mediated resistance genes, is an important mechanism of reduced susceptibility to quinolones. This study’s findings illustrate the *qnr*B gene observation rate of 80% between ciprofloxacin-resistant *K. pneumoniae* isolates. Similarly, the plasmid-mediated *qnr*B resistance gene was detected in 29.4% of the tested *K. pneumoniae* isolates [51]. Also, this gene was the third most observed quinolone resistance-associated gene but with a much lower frequency (25.7%) [52]. These *qnr*B variable detection rates could be attributed to the sample size, the presence of diverse Qnr proteins encoding genes that may be affected by geographic distribution, and various criteria of quinolone resistance.

The acquisition of genes encoding certain efflux pumps is usually associated with tetracycline resistance. In this study, the *tet*B gene was observed in only 5/15 (33.3%) of tetracycline-resistant *K. pneumoniae* isolates. Also, a low rate (16.3%; 8/49) of this gene was demonstrated by [53]. This low level of tetracycline resistance among *K. pneumoniae* isolates may be attributed to less use of tetracyclines in hospitals in our study as it is formed only in oral dosage forms which are infrequently used for hospitalized patients, especially in intensive care units. Conversely, the *tet*B gene was recognized by [54] in all of their tested isolates. The *tet*B gene variable observation rates could also be explained by the presence of further *tet* gene variants that may contribute to *tet*B gene negative isolates with phenotypic tetracyclines resistance or the existence of other molecular mechanisms.

The extensive plasmid sharing in *K. pneumoniae*, which causes the acquisition of resistance throughout horizontal gene transmission, could be attributed to the concurrent existence of various resistance genes with subsequently reduced susceptibility to multiple antimicrobial classes. This study demonstrated that 2 isolates simultaneously carried ESβL-, carbapenemase-, aminoglycoside resistance-, quinolone resistance-, and tetracycline resistance-encoding genes. In a related study, two *K. pneumoniae* isolates simultaneously carried ESβLs, AmpC, and carbapenemase genes [39].

## 5. Conclusions

A relatively high incidence of biofilm formation and MDR phenotypes were observed among *K. pneumoniae* isolates. The *fim*D and *mrk*D genes encoding adhesins were frequently identified in these isolates. Moreover, an elevated percent of the isolates were ESβL producers and carried *bla*_SHV_ with or without the *bla*_CTX-M_ gene. Additionally, the *bla*_NDM_ was the major carbapenemase encoding gene detected in CRKp isolates. An elevated prevalence of *aac(3)IIa* and *qnr*B genes amongst aminoglycosides and ciprofloxacin-resistant *K. pneumoniae* isolates, respectively, was observed. So, the uncontrolled overuse/misuse of antibiotics complicates the problem of MDR bacteria to many of the antimicrobial agents available worldwide leading to limited therapeutic options. Therefore, the findings specified the necessity for forthcoming experimental investigations that warranted to elucidate the underlying mechanisms of AMR and biofilm formation in *K. pneumoniae* that are associated with transferable genetic elements to implement effective infection control measures and find an efficient treatment of this superbug.

## Figures and Tables

**Table 3:** Antimicrobial susceptibility profiles of *K. pneumoniae* isolates.

Antimicrobial Agents	*K. pneumoniae* (N = 63)		
	Susceptible (%)	Intermediate Resistant (%)	Resistant (%)
Ceftriaxone	1 (1.6)	9 (14.3)	53 (84.1)
Cefotaxime	0 (0)	11 (17.5)	52 (82.5)
Ceftazidime	7 (11.1)	12 (19.1)	44 (69.8)
Meropenem	7 (11.1)	22 (34.9)	34 (54)
Amoxicillin/Clavulanic	2 (3.2)	11 (17.5)	50 (79.3)
Amikacin	32 (50.8)	12 (19.1)	19 (30.1)
Gentamycin	16 (25.4)	21 (33.3)	26 (41.3)
Doxycycline	24 (38.1)	24 (38.1)	15 (23.8)
Tetracycline	22 (34.9)	28 (44.4)	13 (20.7)
Ciprofloxacin	13 (20.7)	20 (31.7)	30 (47.6)

**Table 4:** The co-existence of plasmid-mediated AMR-associated genes in *K. pneumoniae* isolates.

Category	Plasmid-Mediated AMR Genes					No. of Isolates (%)	Total No. of Isolates (%)
	EsβLs *	Carbapenemase *	*aac(3)IIa*	*qnr*B	*tet*B		
A	+ *	+	+	+	+	2 (3.2)	2 (3.2)
B	+	+	+	+	− *	9 (14.3)	11 (17.4)
	+	+	+	−	+	2 (3.2)	
C	+	+	+	−	−	6 (9.5)	22 (35)
	+	+	−	+	−	14 (22.2)	
	+	+	−	−	+	1 (1.6)	
	+	−	+	+	−	1 (1.6)	
D	+	+	−	−	−	11 (17.4)	17 (27)
	+	−	+	−	-	3 (4.8)	
	+	−	−	+	−	2 (3.2)	
	−	−	+	+	−	1 (1.6)	
E	+	−	−	−	−	11 (17.4)	11 (17.4)

* EsβLs, *bla*_SHV_ or *bla*_CTX-M_; carbapenemase, *bla*_KPC,_ *bla*_OXA-48-like,_ or *bla*_NDM_; +, presence of the corresponding gene; −, absence of the corresponding gene.

## Data Availability

The original contributions presented in the study are included in the article, further inquiries can be directed to the corresponding author/s.
